# Comparison of CATs, CURB-65 and PMEWS as Triage Tools in Pandemic Influenza Admissions to UK Hospitals: Case Control Analysis Using Retrospective Data

**DOI:** 10.1371/journal.pone.0034428

**Published:** 2012-04-03

**Authors:** Puja R. Myles, Jonathan S. Nguyen-Van-Tam, Wei Shen Lim, Karl G. Nicholson, Stephen J. Brett, Joanne E. Enstone, James McMenamin, Peter J. M. Openshaw, Robert C. Read, Bruce L. Taylor, Barbara Bannister, Malcolm G. Semple

**Affiliations:** 1 Division of Epidemiology and Public Health, University of Nottingham, Nottingham, United Kingdom; 2 Department of Respiratory Medicine, Nottingham University Hospitals NHS Trust, Nottingham, United Kingdom; 3 Infectious Diseases Unit, University Hospitals of Leicester NHS Trust, Leicester Royal Infirmary, Leicester, United Kingdom; 4 Centre for Peri-operative Medicine and Critical Care Research, Imperial College Healthcare NHS Trust, London, United Kingdom; 5 Health Protection Scotland, NHS National Services Scotland, Glasgow, United Kingdom; 6 Centre for Respiratory Infections, National Heart and Lung Institute, Imperial College, London, United Kingdom; 7 Department of Infection & Immunity, University of Sheffield, Royal Hallamshire Hospital, Sheffield, United Kingdom; 8 Department of Critical Care, Portsmouth Hospitals NHS Trust, Portsmouth, United Kingdom; 9 Infectious Diseases Department, Royal Free Hampstead NHS Trust, London, United Kingdom; 10 Department of Women's and Children's Health, Institute of Translational Medicine, University of Liverpool, Liverpool, United Kingdom; University of Calgary & ProvLab Alberta, Canada

## Abstract

Triage tools have an important role in pandemics to identify those most likely to benefit from higher levels of care. We compared Community Assessment Tools (CATs), the CURB-65 score, and the Pandemic Medical Early Warning Score (PMEWS); to predict higher levels of care (high dependency - Level 2 or intensive care - Level 3) and/or death in patients at or shortly after admission to hospital with A/H1N1 2009 pandemic influenza. This was a case-control analysis using retrospectively collected data from the FLU-CIN cohort (1040 adults, 480 children) with PCR-confirmed A/H1N1 2009 influenza. Area under receiver operator curves (AUROC), sensitivity, specificity, positive predictive values and negative predictive values were calculated. CATs best predicted Level 2/3 admissions in both adults [AUROC (95% CI): CATs 0.77 (0.73, 0.80); CURB-65 0.68 (0.64, 0.72); PMEWS 0.68 (0.64, 0.73), p<0.001] and children [AUROC: CATs 0.74 (0.68, 0.80); CURB-65 0.52 (0.46, 0.59); PMEWS 0.69 (0.62, 0.75), p<0.001]. CURB-65 and CATs were similar in predicting death in adults with both performing better than PMEWS; and CATs best predicted death in children. CATs were the best predictor of Level 2/3 care and/or death for both adults and children. CATs are potentially useful triage tools for predicting need for higher levels of care and/or mortality in patients of all ages.

## Introduction

Triage tools identifying need for higher levels of care and risk of severe outcome have an important role in pandemic situations where secondary care capacity may be insufficient to meet demand [1]. The time available for clinical decision making may be limited by workload pressures and healthcare workers unfamiliar with clinical assessment and admission decision making may be asked to fulfil ‘gatekeeper’ roles. The CURB-65 score is a validated predictor of 30-day mortality from community acquired pneumonia in adults but was never intended for use in children [2], [3]. The CURB-65 score does not perform as well in predicting higher levels of care and was not designed to predict mortality from non-pneumonic presentations [4], [5]. Challen et al proposed the Pandemic Medical Early Warning Score (PMEWS) as a clinical triage tool to aid hospital admission decisions for adults in a pandemic situation [6]. They validated PMEWS in adults presenting to hospital with community acquired pneumonia and found that it was better than the CURB-65 score for predicting need for admission and higher levels of care but had limited ability to predict mortality.

In 2009, the Department of Health England published a package of care that included Community Assessment Tools (CATs) and patient pathways for use by the NHS in a severe pandemic event [7]. CATs were developed to help non-specialist front-line staff identify which sick children and adults are most likely to benefit from interventions and levels of care only available in hospitals when resources are limited. CATs use six objective and one subjective criteria based on simple clinical assessment. Meeting any CATs criterion warrants referral and admission to hospital. Criteria are:

- Severe respiratory distress,
- Increased respiratory rate,
- Oxygen saturation ≤92% on pulse oximetry breathing air, or on oxygen,
- Respiratory exhaustion,
- Severe dehydration or shock,
- Altered consciousness level and
- Causing other clinical concern.

While criterion fields are common to adult and paediatric CATs, the abnormal physiological thresholds and clinical signs are age-appropriate. Like PMEWS, there is no requirement for laboratory investigation to complete the assessment. CATs were only intended for use “during severe and exceptional circumstances when surge demand for healthcare services leads to a need for strict triage”; and as such, were not deployed during the 2009/10 pandemic.

Goodacre and colleagues (2010) conducted an evaluation of the discriminatory value of the CURB-65 score, PMEWS and CATs for predicting severe illness or mortality in patients with suspected pandemic influenza, but were unable to draw any conclusions regarding their clinical utility in a pandemic situation due to insufficient case numbers especially of adults, and a low incidence of severe outcome [8]. We aimed to use data from the much larger Influenza Clinical Information Network (FLU-CIN) cohort to compare the clinical validity and utility of CATs, CURB-65 and PMEWS as predictors for higher levels of care, in-patient mortality and severe combined outcome in pandemic influenza.

## Methods

FLU-CIN was an ‘emergency’ surveillance network established by the Department of Health England. FLU-CIN used a purposive sampling frame based on 13 sentinel hospitals situated in five clinical ‘hubs’ in Nottingham, Leicester, London, Sheffield and Liverpool, with contributions from a further 45 non-sentinel hospitals in England and 17 in Scotland, Wales and Northern Ireland. Between April 2009 and January 2010, clinical, epidemiological and outcome data were collected on 1520 patients (800 female, 480 children <16 years) admitted to participating UK hospitals with confirmed A/H1N1 2009 influenza infection.

The details of data collection and the findings have been described elsewhere [9]. A/H1N1 2009 influenza infection was diagnosed by a positive reverse transcribed polymerase chain reaction (PCR) result from respiratory samples obtained during the admission episode. Data was gathered from routine case notes using the first recorded routine clinical assessment on or shortly after admission. A case-controlled analysis using retrospective data of the predictive ability of CATs, CURB-65 and PMEWS was conducted using the full FLU-CIN cohort. Analyses were conducted by age group. A complete case analysis was used.

CATs scores were calculated by awarding a single point for each of the following: severe respiratory distress, increased respiratory rate, oxygen saturation ≤92% (in air or supplemental oxygen), respiratory exhaustion, severe clinical dehydration, altered consciousness and a maximum of one point for causing any other clinical concern to the attending clinicians; on or shortly after admission to hospital. The definitions for CATs criteria differ for children and adults and are provided in Appendix S1. CURB-65 scores were calculated by awarding one point for each of the following: confusion, urea >7 mmol/l, respiratory rate ≥30/minute, low systolic (<90 mmHg) or diastolic (≤60 mmHg) blood pressure and age ≥65 years [2]. PMEWS scores were calculated using the algorithm described by Challen et al. with points being allocated on a weighted basis for varying values of the following indicators: respiratory rate, oxygen saturation, heart rate, systolic blood pressure, temperature, neurological signs (level of alertness). In addition, a point was awarded for age ≥65 years, social isolation, chronic disease and performance status of limited activity (modified Karnofsky >2) [6].

The discriminatory value of the three tools was initially compared using logistic regression to assess whether various outcomes: patients admitted to higher levels of care (high dependency care - Level 2 or intensive care - Level 3), death, or severe outcomes as a whole (a combined measure indicating either Level 2/3 admission or death); were more likely to have higher scores than controls. Each scoring system was included in a univariable logistic model as a continuous variable on the assumption that the scores would follow a linear trend.

Results were presented as unadjusted Odds Ratios (ORs) and 95 per cent Confidence Intervals (95% CI). The resulting ORs could therefore be interpreted as the increased likelihood of a given clinical outcome for every unit increase on the scoring scale.

The three tools were then compared on their ability to predict: admission to higher levels of care, death or severe outcome (combined higher level of care and or death); using area under the Receiver Operating Characteristic (ROC) curve (AUROC) comparisons with 95% confidence intervals. Calibration of the model was tested using the Hosmer-Lemeshow goodness-of-fit test.

The sensitivity (the proportion of true positives that are correctly identified by the test), specificity (the proportion of true negatives that were correctly predicted by the test), positive predictive value (PPV) i.e., the proportion of test positive patients who actually had the outcome; and negative predictive value (NPV) i.e., the proportion of test negative patients who were actually negative for the outcome, were calculated for each of the tools using various score thresholds. All analyses were carried out using Stata version 11.0 (StataCorp. 2009).

Before commencement, FLU-CIN procedures were reviewed by the Ethics and Confidentiality Committee of the National Information Governance Board for Health and Social Care in England and approved for collection, storage and use of personal data for surveillance purposes.

## Results

The study sample comprised 1040 (68.4%) adults and 480 (31.6%) children (age<16 years) admitted to hospital in two pandemic waves: Spring/Summer 2009 (n = 601) and Autumn/Winter 2009/10 (n = 919). The median age was 26 years (interquartile range 9 to 44 years). There were 800 (52.6%) females of whom 83 aged 14 to 44 years were pregnant (20.8%). The clinical characteristics of the first-wave cohort have been described previously [9]. Tables 1 and 2 present the distribution of CATs scores, CURB-65 scores and PMEWS scores by admission to higher levels of care and mortality. Results are presented as unadjusted Odds Ratios (ORs) and 95% Confidence Intervals (95%CI). The resulting ORs could therefore be interpreted as the increased likelihood of a given clinical outcome for every unit increase on each scoring scale. For each of the triage tools, adult patients with any severe outcome (higher level of care and/or in-patient death) were more likely to have higher scores as compared to controls. In children, both CATs and PMEWS scores were more likely to be higher in patients with severe outcomes.

**Table 1:** Distribution of CATs, CURB-65 and PMEWS scores according to outcome measures (Level 2/3 admission, mortality, combined measure of severe outcomes) in adults (≥16 years).

Triage tool	Level 2 or 3 admission		Death		Combined severe outcomes*	
	Yes (n = 177)	No (n = 863)	Yes (n = 62)	No (n = 978)	Yes (n = 191)	No (n = 849)
**CATs scores**						
0	10 (5.7%)	224 (26.0%)	6 (9.7%)	228 (23.3%)	12 (6.3%)	222 (26.2%)
1	27 (15.3%)	306 (35.5%)	10 (16.1%)	323 (33.0%)	30 (15.7%)	303 (35.7%)
2	54 (30.5%)	223 (25.8%)	17 (27.4%)	260 (26.6%)	57 (26.7%)	220 (25.9%)
3	47 (26.6%)	96 (11.1%)	17 (27.4%)	126 (12.9%)	51 (26.7%)	92 (10.8%)
4	31 (17.5%)	14 (1.6%)	10 (16.1%)	35 (3.6%)	33 (17.3%)	12 (1.4%)
5	5 (2.8%)	0 (0.0%)	1 (1.6%)	4 (0.4%)	5 (2.6%)	0 (0.0%)
6	1 (0.6%)	0 (0.0%)	0 (0.0%)	1 (0.1%)	1 (0.5%)	0 (0.0%)
7	2 (1.1%)	0 (0.0%)	1 (1.6%)	1 (0.1%)	2 (1.1%)	0 (0.0%)
Unadjusted OR (95% CI)	4.61 (3.45, 6.16); p trend<0.001		2.83 (1.91, 4.19); p trend<0.001		4.57 (3.44, 6.07); p trend<0.001	
**CURB-65 scores**						
0	32 (18.1%)	380 (44.0%)	6 (9.7%)	406 (41.5%)	33 (17.3%)	379 (44.6%)
1	70 (39.6%)	321 (37.2%)	26 (41.9%)	365 (37.3%)	75 (39.3%)	316 (37.2%)
2	50 (28.3%)	138 (16.0%)	22 (35.5%)	166 (17.0%)	56 (29.3%)	132 (15.6%)
3	24 (13.6%)	24 (2.8%)	8 (12.9%)	40 (4.1%)	26 (13.6%)	22 (2.6%)
4	1 (0.6%)	0 (0.0%)	0 (0.0%)	1 (0.1%)	1 (0.5%)	0 (0.0%)
Unadjusted OR (95% CI)	2.15 (1.79, 2.59); p trend<0.001		2.20 (1.68, 2.90); p trend<0.001		2.26 (1.89, 2.72); p trend<0.001	
**PMEWS scores**						
0	0 (0.0%)	1 (0.1%)	0 (0.0%)	1 (0.1%)	0 (0.0%)	1 (0.1%)
1	4 (2.3%)	53 (6.1%)	1 (1.6%)	56 (5.7%)	4 (2.1%)	53 (6.2%)
2	13 (7.3%)	96 (11.1%)	7 (11.3%)	102 (10.4%)	16 (8.4%)	93 (11.0%)
3	10 (5.7%)	131 (15.2%)	6 (9.7%)	135 (13.8%)	12 (6.3%)	129 (15.2%)
4	15 (8.5%)	125 (14.5%)	7 (11.3%)	133 (13.6%)	16 (8.4%)	124 (14.6%)
5	12 (6.8%)	130 (15.1%)	2 (3.2%)	140 (14.3%)	13 (6.8%)	129 (15.2%)
6	25 (14.1%)	110 (12.8%)	10 (16.1%)	125 (12.8%)	28 (14.7%)	107 (12.6%)
7	31 (17.5%)	79 (9.2%)	12 (19.4%)	98 (10.0%)	33 (17.3%)	77 (9.1%)
8	23 (13.0%)	49 (5.7%)	4 (6.5%)	68 (7.0%)	24 (12.6%)	48 (5.7%)
9	20 (11.3%)	58 (6.7%)	7 (11.3%)	71 (7.3%)	20 (10.5%)	58 (6.8%)
10	15 (8.5%)	20 (2.3%)	4 (6.5%)	31 (3.2%)	15 (7.9%)	20 (2.4%)
11	5 (2.8%)	8 (0.9%)	1 (1.6%)	12 (1.2%)	6 (3.1%)	7 (0.8%)
≥12	4 (2.3%)	3 (0.4%)	1 (1.6%)	6 (0.6%)	4 (2.1%)	3 (0.4%)
Unadjusted OR (95% CI)	1.29 (1.21, 1.38); p trend<0.001		1.14 (1.03, 1.26); p trend = 0.009		1.27 (1.19, 1.36); p trend<0.001	

* Combined measure of severe outcomes (Level 2/3 admission or death).

**Table 2:** Distribution of CATs, CURB-65 and PMEWS scores according to outcome measures (Level 2/3 admission, mortality, combined measure of severe outcomes) in children (<16 years).

Triage tool	Level 2 or 3 admission		Death		Combined severe outcomes*	
	Yes (n = 73)	No (n = 407)	Yes (n = 18)	No (n = 462)	Yes (n = 77)	No (n = 403)
**CATs scores**						
0	1 (9.6%)	148 (36.4%)	1 (5.6%)	154 (33.3%)	7 (9.1%)	148 (36.7%)
1	18 (24.7%)	151 (37.1%)	3 (16.7%)	166 (35.9%)	18 (23.4%)	151 (37.5%)
2	22 (30.1%)	72 (17.7%)	8 (44.4%)	86 (18.6%)	23 (29.9%)	71 (17.6%)
3	19 (26.0%)	33 (8.1%)	4 (22.2%)	48 (10.4%)	21 (27.3%)	31 (7.7%)
4	5 (6.9%)	2 (0.5%)	1 (5.6%)	6 (1.3%)	5 (6.5%)	2 (0.5%)
5	2 (2.7%)	1 (0.3%)	1 (5.6%)	2 (0.4%)	3 (3.9%)	0 (0.0%)
6	0 (0.0%)	0 (0.0%)	0 (0.0%)	0 (0.0%)	0 (0.0%)	0 (0.0%)
7	0 (0.0%)	0 (0.0%)	0 (0.0%)	0 (0.0%)	0 (0.0%)	0 (0.0%)
Unadjusted OR (95% CI)	3.76 (2.47, 5.71); p trend<0.001		3.18 (1.60, 6.31); p trend = 0.001		4.39 (2.86, 6.72); p trend<0.001	
**CURB-65 scores**						
0	5 (6.9%)	62 (15.2%)	1 (5.6%)	66 (14.3%)	5 (6.5%)	62 (15.4%)
1	38 (52.1%)	169 (41.5%)	10 (55.6%)	197 (42.6%)	40 (52.0%)	167 (41.4%)
2	25 (34.3%)	166 (40.8%)	6 (33.3%)	185 (40.0%)	27 (35.1%)	164 (40.7%)
3	5 (6.9%)	10 (2.5%)	1 (5.6%)	14 (3.0%)	5 (6.5%)	10 (2.5%)
4	0 (0.0%)	0 (0.0%)	0 (0.0%)	0 (0.0%)	0 (0.0%)	0 (0.0%)
Unadjusted OR (95% CI)	1.21 (0.86, 1.70); p trend = 0.264		1.14 (0.60, 2.14); p trend = 0.694		1.23 (0.88, 1.71); p = 0.226	
**PMEWS scores**						
0	0 (0.0%)	0 (0.0%)	0 (0.0%)	0 (0.0%)	0 (0.0%)	0 (0.0%)
1	0 (0.0%)	8 (2.0%)	0 (0.0%)	8 (1.7%)	0 (0.0%)	8 (2.0%)
2	0 (0.0%)	16 (3.9%)	0 (0.0%)	16 (3.5%)	0 (0.0%)	16 (4.0%)
3	1 (1.4%)	14 (3.4%)	0 (0.0%)	15 (3.3%)	1 (1.3%)	14 (3.5%)
4	3 (4.1%)	24 (5.9%)	1 (5.6%)	26 (5.6%)	3 (3.9%)	24 (6.0%)
5	2 (2.7%)	31 (7.6%)	1 (5.6%)	32 (6.9%)	2 (2.6%)	31 (7.7%)
6	4 (5.5%)	51 (12.5%)	0 (0.0%)	55 (11.9%)	4 (5.2%)	51 (12.7%)
7	12 (16.4%)	67 (16.5%)	4 (22.2%)	75 (16.2%)	13 (16.9%)	66 (16.4%)
8	11 (15.1%)	70 (17.2%)	1 (5.6%)	80 (17.3%)	11 (14.3%)	70 (17.4%)
9	17 (23.3%)	93 (22.9%)	4 (22.2%)	106 (22.9%)	19 (24.7%)	91 (22.6%)
10	5 (6.9%)	24 (5.9%)	2 (11.1%)	27 (5.8%)	6 (7.8%)	23 (5.7%)
11	8 (11.0%)	6 (1.5%)	1 (5.6%)	13 (2.8%)	8 (10.4%)	6 (1.5%)
≥12	10 (13.7%)	3 (0.7%)	4 (22.2%)	9 (2.0%)	10 (13.0%)	3 (0.7%)
Unadjusted OR (95% CI)	1.47 (1.27, 1.69); p trend<0.001		1.48 (1.15, 1.91); p trend = 0.003		1.48 (1.29, 1.70); p trend<0.001	

* Combined measure of severe outcomes (Level 2/3 admission or death).

Calibration i.e. the proximity of observed and expected values or goodness-of-fit of the logistic regression models was tested using the Hosmer-Lemeshow goodness-of-fit test. In adults, the outcomes ‘Level 2 or 3 admission’ and ‘Death’, all logistic regression models for all three triage tools (CATs, CURB-65 and PMEWS) showed good calibration. When considering combined severe outcomes (Level 2/3 admission or death), only CATs and CURB-65 demonstrated good calibration between observed and expected values; PMEWS had a poor fit (p = 0.0453). In children, CATs was the only triage tool for which the logistic regression model showed good calibration for all three outcomes. Both CURB-65 and PMEWS showed good calibration between observed and expected values for ‘death’ but poor calibration when used for predicting ‘Level 2 or 3 admission’ (p = 0.0204 and p = 0.0176 respectively).

**Figure 1:**
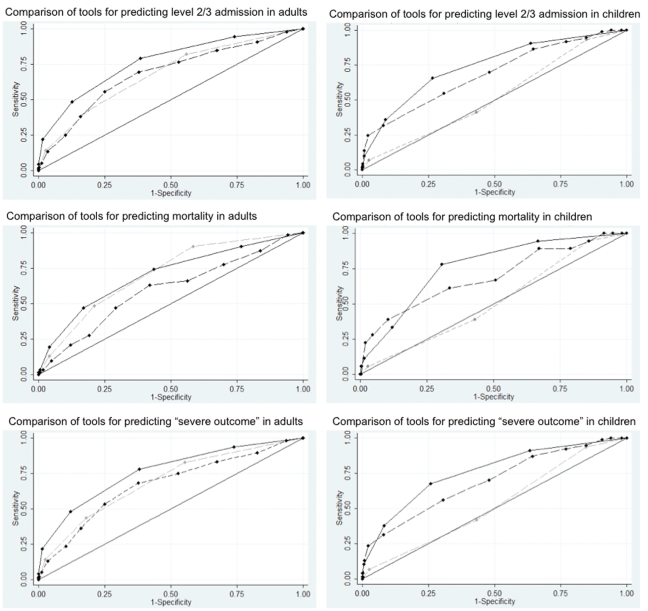
The predictive values of CATs, CURB-65 and PMEWS for predicting severe outcomes in adults and children with pandemic influenza. ROC curves comparing the predictive value of CATs (black solid line), CURB-65 (grey dash line) and PMEWS (black dash line) in relation to Level 2/3 admissions (upper panels), mortality (middle panels) and combined severe outcomes (lower panels) in adults (left panels, age ≥16 years, n = 1040) and children (right panels, age<16 years, n = 480).

The ROC curves and AUROC values comparing the predictive value of the three clinical triage tools are described in figure 1. CATs showed the best predictive performance for Level 2/3 admissions in both adults [AUROC (95% CI): CATs 0.77 (0.73, 0.80); CURB-65 0.68 (0.64, 0.72); PMEWS 0.68 (0.64, 0.73), comparison of AUROCs p<0.001, n = 1040] and children [AUROC (95% CI): CATs 0.74 (0.68, 0.80); CURB-65 0.52 (0.46, 0.59); PMEWS 0.69 (0.62, 0.75), p<0.001, n = 480].

CURB-65 and CATs had similar performance in predicting in-patient mortality in adults [AUROC (95% CI): CATs 0.70 (0.63, 0.77); CURB-65 0.71 (0.65, 0.77); PMEWS 0.60 (0.52, 0.67), p = 0.009] but CATs performed best as a predictor of mortality in children [AUROC (95% CI): CATs 0.76 (0.66, 0.86); CURB-65 0.51 (0.39, 0.63); PMEWS 0.69 (0.55, 0.83), p = 0.002].

CATs were the best predictor of severe outcome defined as a combined measure of either Level 2/3 admission or in-patient death; for both adults [AUROC (95% CI): CATs 0.76 (0.73, 0.80); CURB-65 0.69 (0.65, 0.72); PMEWS 0.67 (0.63, 0.71), p<0.001] and children [AUROC (95% CI): CATs 0.76 (0.70, 0.82); CURB-65 0.53 (0.46, 0.59); PMEWS 0.69 (0.63, 0.76), p<0.001].

In a sensitivity analysis restricted to adults with A/H1N1 2009 and a diagnosis of pneumonia validated by radiographic reports; the adult CATs was the best predictor of Level 2/3 admission [AUROC (95% CI): CATs 0.78 (0.72, 0.83); CURB-65 0.70 (0.63, 0.77); PMEWS 0.70 (0.6, 0.76), p = 0.034]. CURB-65 and adult CATs were similar and better than PMEWS in predicting in-patient death [AUROC (95% CI): CURB-65 0.73 (0.63, 0.82); CATs 0.66 (0.56, 0.76); PMEWS 0.58 (0.46, 0.69), p = 0.038]. The adult CATs was the best predictor of severe outcome [AUROC (95% CI): CATs 0.77 (0.71, 0.83); CURB-65 0.71 (0.65, 0.78); PMEWS 0.68 (0.61, 0.74), p = 0.027].

Tables 3 and 4 explore the clinical utility of various threshold scores for each of the triage tools. In adults, a CATs score ≥3 was the best predictor of Level 2/3 admissions or in-patient death or combined severe outcome when compared to various cut-off scores for either CURB-65 or PMEWS. In children, a CATs score ≥3 was the best predictor of Level 2/3 admission and combined severe outcome; performing marginally better than a PMEWS score >9, both significantly better than CURB-65. In children, a PMEWS score >9 was the best predictor of mortality in children; performing marginally better than a CATs score >3, both significantly better than CURB-65.

**Table 3:** Predictive values of CATs, CURB-65 and PMEWS scores for predicting severe outcomes in adults (≥16 years, n = 1040).

Outcome	Score	ROC area (95%CI)	Sensitivity % (95%CI)	Specificity % (95%CI)	PPV % (95%CI)	NPV % (95%CI)
**Level 2/3 admission**	CURB-65≥2	0.62 (0.58, 0.66)	42.4 (35.0, 50.0)	81.2 (78.5, 83.8)	31.6 (25.8, 38.0)	87.3 (84.8, 89.5)
	CURB-65≥3	0.56 (0.53, 0.58)	14.1 (9.4, 20.1)	97.2 (95.9, 98.2)	51.0 (36.3, 65.6)	84.7 (82.3, 86.9)
	PMEWS>1	0.52 (0.51, 0.53)	97.7 (94.3, 99.4)	6.3 (4.7, 8.1)	17.6 (15.3, 20.1)	93.1 (83.3, 98.1)
	PMEWS>2	0.54 (0.51, 0.56)	90.4 (85.1, 94.3)	17.4 (14.9, 20.1)	18.3 (15.8, 21.1)	89.8 (84.2, 94.0)
	PMEWS>3	0.54 (0.51, 0.56)	90.4 (85.1, 94.3)	17.4 (14.9, 20.1)	18.3 (15.8, 21.1)	89.8 (84.2, 94.0)
	PMEWS>4	0.59 (0.56, 0.62)	84.7 (78.6, 89.7)	32.6 (29.4, 35.8)	20.5 (17.6, 23.6)	91.2 (87.5, 94.1)
	PMEWS>5	0.62 (0.58, 0.65)	76.3 (69.3, 82.3)	47.0 (43.7, 50.4)	22.8 (19.5, 26.4)	90.6 (87.5, 93.2)
	PMEWS>7	0.65 (0.61, 0.69)	55.4 (47.7, 62.8)	74.9 (71.8, 77.7)	31.1 (26.0, 36.5)	89.1 (86.6, 91.3)
	PMEWS>9	0.57 (0.54, 0.61)	24.9 (18.7, 31.9)	89.7 (87.5, 91.6)	33.1 (25.2, 41.8)	85.3 (82.9, 87.6)
	PMEWS>11	0.52 (0.50, 0.54)	5.1 (2.4, 9.4)	98.7 (97.7, 99.4)	45.0 (23.1, 68.5)	83.5 (81.1, 85.8)
	CATs≥3	0.68 (0.64, 0.72)	48.6 (41.0, 56.2)	87.3 (84.8, 89.4)	43.9 (36.8, 51.1)	89.2 (86.9, 91.2)
	CATs≥4	0.60 (0.57, 0.63)	22.0 (16.2, 28.9)	98.4 (97.3, 99.1)	73.6 (59.7, 84.7)	86.0 (83.7, 88.1)
	CATs≥5	0.52 (0.51, 0.54)	4.5 (2.0, 8.7)	100.0 (99.6, 100.0)	100.0 (63.1, 100.0)	83.6 (81.2, 85.8)
**Death**	CURB-65≥2	0.64 (0.57, 0.70)	48.4 (35.5, 61.4)	78.8 (76.1, 81.4)	12.7 (8.7, 17.6)	96.0 (94.4, 97.3)
	CURB-65≥3	0.54 (0.50, 0.59)	12.9 (5.7, 23.9)	95.8 (94.4, 97.0)	16.3 (7.3, 29.7)	94.6 (92.9, 95.9)
	PMEWS>1	0.52 (0.50, 0.54)	98.4 (91.3, 100.0)	5.8 (4.4, 7.5)	6.2 (4.8, 7.9)	98.3 (90.8, 100.0)
	PMEWS>2	0.52 (0.47, 0.56)	87.1 (76.1, 94.3)	16.3 (14.0, 18.7)	6.2 (4.7, 8.0)	95.2 (90.8, 97.9)
	PMEWS>3	0.52 (0.47, 0.56)	87.1 (76.1, 94.3)	16.3 (14.0, 18.7)	6.2 (4.7, 8.0)	95.2 (90.8, 97.9)
	PMEWS>4	0.54 (0.48, 0.59)	77.4 (65.0, 87.1)	30.1 (27.2, 33.0)	6.6 (4.9, 8.6)	95.5 (92.5, 97.5)
	PMEWS>5	0.55 (0.49, 0.61)	66.1 (53.0, 77.7)	43.7 (40.5, 46.8)	6.9 (5.0, 9.3)	95.3 (92.9, 97.1)
	PMEWS>7	0.59 (0.52, 0.65)	46.8 (34.0, 59.9)	70.8 (67.8, 73.6)	9.2 (6.3, 13.0)	95.4 (93.7, 96.8)
	PMEWS>9	0.54 (0.49, 0.60)	21.0 (11.7, 33.2)	87.7 (85.5, 89.7)	9.8 (5.3, 16.1)	94.6 (92.9, 96.0)
	PMEWS>11	0.51 (0.48, 0.53)	3.2 (0.4, 11.2)	98.2 (97.1, 98.9)	10.0 (1.2, 31.7)	94.1 (92.5, 95.5)
	CATs≥3	0.65 (0.58, 0.71)	46.8 (34.0, 59.9)	82.9 (80.4, 85.2)	14.8 (10.1, 20.6)	96.1 (94.6, 97.3)
	CATs≥4	0.58 (0.53, 0.63)	19.4 (10.4, 31.4)	95.8 (94.4, 97.0)	22.6 (12.3, 36.2)	94.9 (93.4, 96.2)
	CATs≥5	0.51 (0.49, 0.54)	3.2 (0.4, 11.2)	99.4 (98.7, 99.8)	25.0 (3.2, 65.1)	94.2 (92.6, 95.5)
**Combined severe** *	CURB-65≥2	0.63 (0.59, 0.66)	43.5 (36.3, 50.8)	81.9 (79.1, 84.4)	35.0 (29.0, 41.5)	86.6 (84.0, 88.8)
	CURB-65≥3	0.56 (0.53, 0.58)	14.1 (9.5, 19.9)	97.4 (96.1, 98.4)	55.1 (40.2, 69.3)	83.5 (81.0, 85.7)
	PMEWS>1	0.52 (0.51, 0.53)	97.9 (94.7, 99.4)	6.4 (4.8, 8.2)	19.0 (16.6, 21.6)	93.1 (83.3, 98.1)
	PMEWS>2	0.53 (0.51, 0.56)	89.5 (84.3, 93.5)	17.3 (14.8, 20.0)	19.6 (17.0, 22.4)	88.0 (82.1, 92.5)
	PMEWS>3	0.53 (0.51, 0.56)	89.5 (84.3, 93.5)	17.3 (14.8, 20.0)	19.6 (17.0, 22.4)	88.0 (82.1, 92.5)
	PMEWS>4	0.58 (0.55, 0.61)	83.2 (77.2, 88.2)	32.5 (29.4, 35.8)	21.7 (18.8, 24.9)	89.6 (85.7, 92.8)
	PMEWS>5	0.61 (0.57, 0.65)	74.9 (68.1, 80.9)	47.1 (43.7, 50.5)	24.2 (20.8, 27.8)	89.3 (86.0, 92.0)
	PMEWS>7	0.64 (0.60, 0.68)	53.4 (46.1, 60.6)	74.9 (71.9, 77.8)	32.4 (27.2, 37.9)	87.7 (85.1, 90.0)
	PMEWS>9	0.57 (0.53, 0.60)	23.6 (17.7, 30.2)	89.6 (87.4, 91.6)	33.8 (25.9, 42.5)	83.9 (81.3, 86.2)
	PMEWS>11	0.52 (0.50, 0.54)	5.2 (2.5, 9.4)	98.8 (97.8, 99.4)	50.0 (27.2, 72.8)	82.3 (79.8, 84.6)
	CATs≥3	0.68 (0.64, 0.72)	48.2 (40.9, 55.5)	87.8 (85.4, 89.9)	46.9 (39.8, 54.2)	88.3 (85.9, 90.4)
	CATs≥4	0.60 (0.57, 0.63)	21.5 (15.9, 28.0)	98.6 (97.5, 99.3)	77.4 (63.8, 87.7)	84.8 (82.4, 87.0)
	CATs≥5	0.52 (0.51, 0.54)	4.2 (1.8, 8.1)	100.0 (99.6, 100.0)	100.0 (63.1, 100.0)	82.3 (79.8, 84.6)

* Combined measure of severe outcomes (Level 2/3 admission or death).

**Table 4:** Predictive values of CATs, CURB-65 and PMEWS scores for predicting severe outcomes in children (<16 years, n = 480).

Outcome	Score	ROC area (95%CI)	Sensitivity % (95%CI)	Specificity % (95%CI)	PPV % (95%CI)	NPV % (95%CI)
**Level 2/3 admission**	CURB-65≥2	0.49 (0.43, 0.55)	41.1 (29.7, 53.2)	56.8 (51.8, 61.6)	14.6 (10.0, 20.1)	84.3 (79.4, 88.4)
	CURB-65≥3	0.52 (0.49, 0.55)	6.8 (2.3, 15.3)	97.5 (95.5, 98.8)	33.3 (11.8, 61.6)	85.4 (81.8, 88.5)
	PMEWS>1	0.51 (0.50, 0.52)	100.0 (95.1, 100.0)	2.0 (0.9, 3.8)	15.5 (12.3, 19.0)	100.0 (63.1, 100.0)
	PMEWS>2	0.53 (0.52, 0.54)	100.0 (95.1, 100.0)	5.9 (3.8, 8.6)	16.0 (12.8, 19.7)	100.0 (85.8, 100.0)
	PMEWS>3	0.53 (0.52, 0.54)	100.0 (95.1, 100.0)	5.9 (3.8, 8.6)	16.0 (12.8, 19.7)	100.0 (85.8, 100.0)
	PMEWS>4	0.54 (0.52, 0.56)	98.6 (92.6, 100.0)	9.3 (6.7, 12.6)	16.3 (13.0, 20.1)	97.4 (86.5, 99.9)
	PMEWS>5	0.55 (0.52, 0.58)	94.5 (86.6, 98.5)	15.2 (11.9, 19.1)	16.7 (13.2, 20.6)	93.9 (85.2, 98.3)
	PMEWS>7	0.61 (0.56, 0.65)	86.3 (76.2, 93.2)	35.4 (30.7, 40.2)	19.3 (15.2, 24.0)	93.5 (88.4, 96.8)
	PMEWS>9	0.62 (0.56, 0.68)	54.8 (42.7, 66.5)	69.0 (64.3, 73.5)	24.1 (17.8, 31.3)	89.5 (85.6, 92.7)
	PMEWS>11	0.61 (0.56, 0.66)	24.7 (15.3, 36.1)	97.8 (95.8, 99.0)	66.7 (46.0, 83.5)	87.9 (84.5, 90.7)
	CATs≥3	0.63 (0.58, 0.69)	35.6 (24.7, 47.7)	91.2 (88.0, 93.7)	41.9 (29.5, 55.2)	88.8 (85.3, 91.6)
	CATs≥4	0.54 (0.51, 0.58)	9.6 (3.9, 18.8)	99.3 (97.9, 99.8)	70.0 (34.8, 93.3)	86.0 (82.5, 89.0)
	CATs≥5	0.51 (0.49, 0.53)	2.7 (0.3, 9.5)	99.8 (98.6, 100.0)	66.7 (9.4, 99.2)	85.1 (81.6, 88.2)
**Death**	CURB-65≥2	0.48 (0.36, 0.60)	38.9 (17.3, 64.3)	56.9 (52.3, 61.5)	3.4 (1.4, 6.9)	96.0 (92.9, 98.0)
	CURB-65≥3	0.51 (0.46, 0.57)	5.6 (0.1, 27.3)	97.0 (95.0, 98.3)	6.7 (0.2, 31.9)	96.3 (94.2, 97.9)
	PMEWS>1	0.51 (0.50, 0.51)	100.0 (81.5, 100.0)	1.7 (0.8, 3.4)	3.8 (2.3, 6.0)	100.0 (63.1, 100.0)
	PMEWS>2	0.53 (0.52, 0.54)	100.0 (81.5, 100.0)	5.2 (3.4, 7.6)	3.9 (2.4, 6.2)	100.0 (85.8, 100.0)
	PMEWS>3	0.53 (0.52, 0.54)	100.0 (81.5, 100.0)	5.2 (3.4, 7.6)	3.9 (2.4, 6.2)	100.0 (85.8, 100.0)
	PMEWS>4	0.54 (0.53, 0.55)	100.0 (81.5, 100.0)	8.4 (6.1, 11.4)	4.1 (2.4, 6.4)	100.0 (91.0, 100.0)
	PMEWS>5	0.54 (0.49, 0.60)	94.4 (72.7, 99.9)	14.1 (11.0, 17.6)	4.1 (2.4, 6.5)	98.5 (91.8, 100.0)
	PMEWS>7	0.61 (0.53, 0.69)	88.9 (65.3, 98.6)	32.9 (28.6, 37.4)	4.9 (2.8, 7.8)	98.7 (95.4, 99.8)
	PMEWS>9	0.64 (0.52, 0.76)	61.1 (35.7, 82.7)	66.5 (61.9, 70.7)	6.6 (3.4, 11.5)	97.8 (95.5, 99.1)
	PMEWS>11	0.62 (0.51, 0.72)	27.8 (9.7, 53.5)	95.2 (92.9, 97.0)	18.5 (6.3, 38.1)	97.1 (95.1, 98.5)
	CATs≥3	0.61 (0.49, 0.72)	33.3 (13.3, 59.0)	87.9 (84.5, 90.7)	9.7 (3.6, 19.9)	97.1 (95.0, 98.5)
	CATs≥4	0.55 (0.47, 0.62)	11.1 (1.4, 34.7)	98.3 (96.6, 99.2)	20.0 (2.5, 55.6)	96.6 (94.5, 98.0)
	CATs≥5	0.53 (0.47, 0.58)	5.6 (0.1, 27.3)	99.6 (98.4, 99.9)	33.3 (0.8, 90.6)	96.4 (94.4, 97.9)
**Combined severe** *	CURB-65≥2	0.49 (0.43, 0.55)	41.6 (30.4, 53.4)	56.8 (51.8, 61.7)	15.5 (10.9, 21.2)	83.6 (78.6, 87.8)
	CURB-65≥3	0.52 (0.49, 0.55)	6.5 (2.1, 14.5)	97.5 (95.5, 98.8)	33.3 (11.8, 61.6)	84.5 (80.9, 87.7)
	PMEWS>1	0.51 (0.50, 0.52)	100.0 (95.3, 100.0)	2.0 (0.9, 3.9)	16.3 (13.1, 20.0)	100.0 (63.1, 100.0)
	PMEWS>2	0.53 (0.52, 0.54)	100.0 (95.3, 100.0)	6.0 (3.9, 8.7)	16.9 (13.6, 20.6)	100.0 (85.8, 100.0)
	PMEWS>3	0.53 (0.52, 0.54)	100.0 (95.3, 100.0)	6.0 (3.9, 8.7)	16.9 (13.6, 20.6)	100.0 (85.8, 100.0)
	PMEWS>4	0.54 (0.52, 0.56)	98.7 (93.0, 100.0)	9.4 (6.8, 12.7)	17.2 (13.8, 21.1)	97.4 (86.5, 99.9)
	PMEWS>5	0.55 (0.52, 0.58)	94.8 (87.2, 98.6)	15.4 (12.0, 19.3)	17.6 (14.1, 21.7)	93.9 (85.2, 98.3)
	PMEWS>7	0.61 (0.57, 0.66)	87.0 (77.4, 93.6)	35.7 (31.0, 40.6)	20.6 (16.3, 25.4)	93.5 (88.4, 96.8)
	PMEWS>9	0.63 (0.57, 0.69)	55.8 (44.1, 67.2)	69.5 (64.7, 73.9)	25.9 (19.4, 33.3)	89.2 (85.2, 92.4)
	PMEWS>11	0.61 (0.56, 0.65)	23.4 (14.5, 34.4)	97.8 (95.8, 99.0)	66.7 (46.0, 83.5)	87.0 (83.5, 89.9)
	CATs≥3	0.65 (0.59, 0.70)	37.7 (26.9, 49.4)	91.8 (88.7, 94.3)	46.8 (34.0, 59.9)	88.5 (85.1, 91.4)
	CATs≥4	0.55 (0.51, 0.58)	10.4 (4.6, 19.4)	99.5 (98.2, 99.9)	80.0 (44.4, 97.5)	85.3 (81.8, 88.4)
	CATs≥5	0.52 (0.50, 0.54)	3.9 (0.8, 11.0)	100.0 (99.1, 100.0)	100.0 (29.2, 100.0)	84.5 (80.9, 87.6)

* Combined measure of severe outcomes (Level 2/3 admission or death).

## Discussion

There has been only one head-to-head validation of the performance of CURB-65, PMEWS and CATs during the 2009 pandemic period [8]. Our study has the advantages of large size (n = 1520), confirmation of cases by standardised PCR criteria, and relatively few missing data. Reported cases were followed up without selection and the acquisition of cases closely mirrored the national epidemic curve geographically and temporally [10]. Overall, 16.5% of patients required high dependency or intensive care and 5.3% died.

Two characteristics are crucial when evaluating a clinical prediction test or algorithm: clinical validity and clinical utility. Simon defines clinical validity as the ability of the test result to correlate with a clinical end point or characteristic [11]. Our results show that for each of the three clinical triage tools, a higher score is associated with a greater likelihood of severe clinical outcomes in adult cases, indicating that all three demonstrate clinical validity. In the case of children however, only CATs and PMEWS demonstrate this linear relationship. The ROC curves and AUROC analysis show that in terms of overall performance, CATs are significantly better than CURB-65 or PMEWS as a predictor of combined severe outcomes across all age groups. CURB-65 and CATs are similar in their ability to predict mortality in adults but CATs has better performance in predicting admission to higher levels of care. It can be argued that the latter outcome is more meaningful for clinicians as the primary aim of triage tools is to identify patients who are most likely to benefit from higher levels of care rather than those most likely to die.

The CURB-65 score is validated only for use in adults with community acquired pneumonia to predict 30-day mortality [12], [13]. CURB-65 was not developed for use in non-pneumonic respiratory tract infections nor to predict need for intensive care admission. Results from the current study reinforce these points.

A predictive test has clinical utility only if the use of the test results in improved outcomes for patients [11]. Although clinical utility can only be fully evaluated in a separate prospective cohort, the first step towards this is to determine a suitable threshold value that can discriminate between alternative clinical outcomes. Ideally, a good prediction test should have both high sensitivity and specificity. There is usually a trade-off between sensitivity and specificity. The AUROC provides a combined measure of all the sensitivity/specificity pairs resulting from varying levels of the decision threshold over the entire range of results [14]. There may be some scenarios however, where it is very important not to miss a ‘diagnosis’, one may opt in favour of a higher sensitivity as compared to specificity for e.g. a disease with high mortality where an effective treatment is available [15].

The use of lower thresholds with PMEWS (cut-off values of 1, 2, 3 or 4) demonstrated high sensitivity (77 to 98%) but it is probable that in a pandemic situation where surge capacity is reached, these low thresholds will not offer sufficient discrimination for healthcare prioritisation. Positive predictive values across various thresholds for all scoring systems were generally low but these findings may well reflect the general mildness of 2009 pandemic influenza and the associated low incidence of severe outcomes. As such these measures may not predict the performance of these tools during a more severe influenza pandemic or other highly pathogenic pandemic. Another aspect of clinical utility is the ease of applicability of the test [14]. CURB-65 scores require serum urea measurements which are not easily or rapidly available in community settings and the PMEWS algorithm uses a complex weighted matrix to calculate scores [6]. CATs on the other hand, rely on clinical indicators that can be easily and immediately assessed in community settings and can be repeated and compared in any setting.

The sensitivity analysis restricted to adults with proven A/H1N1 2009 and a diagnosis of community acquired pneumonia validated with reported radiographs shows that in the setting of triage for this pandemic event (and only in this setting), this group of adults would not have been disadvantaged if they were assessed using the adult CATs.

This study shows that on the basis of AUROC values a CATs score ≥3 offers the best predictive value for Level 2/3 admissions and death when considered as independent or combined outcomes in adults. In children, a CATs score ≥3 offers the best predictor of need for higher levels of care and combined severe outcome, while a PMEWS score >9 was marginally the better predictor of mortality, followed closely by a CATs score ≥3. However, as the 95% CI for the two AUROCs overlap, a CATs score ≥3 would offer a reasonable substitute given the overall better performance across age groups for predicting higher levels of care and combined severe outcomes.

A CATs score ≥3 could therefore be used to fast-track patients of any age to critical care earlier in the hope that their survival will improve. In a pandemic situation, when critical care is over-burdened, clinical decision-makers may face very difficult ethical dilemmas concerning access to critical care. CATs allow both children and adults to be triaged within the same conceptual framework. This will be important if scarce resources are to be shared across wider age groups than would occur under normal conditions. The use of CATs scores may help to ensure that treatment access is determined in a fair way, by use of an objective measure of likelihood of benefit from such care. The ethical dilemmas arising in this situation have been considered elsewhere [1].

Appropriate use of triage tools should expedite referral both to hospital, and where scores are high, prompt consideration for admission to Level 2/3 care. This may be associated with improved patient outcomes. A study using the FLU-CIN cohort found that delayed admission to hospital (≥4 days after symptom onset) was significantly associated with increased likelihood of admission to critical care and death [16].

This study confirms the lack of effectiveness of the CURB-65 score as a triage tool for children during an influenza pandemic. The AUROC values for CURB-65 scores in children all approximate to 0.5, not significantly different from pure chance. CURB-65 should not be considered for use in this, and probably any setting involving children.

The validity of the CURB-65 score to predict mortality in adults with A/H1N1 2009 infection both with and without radiograph validated pneumonia is confirmed. Access to laboratory and radiological investigations during a severe pandemic may limit the utility of this tool.

Ideally, the clinical validity and utility of triage tools should be studied prospectively in parallel in a community cohort of pandemic influenza patients, to establish whether they can be used by general practitioners to decide which patients could benefit from hospitalisation.

### Limitations

This was a case-control analysis using retrospectively collected data derived from physicians' first routine clinical assessment of patients during a pandemic event. By design it is not possible to assess intra-observer agreement, inter-observer agreement or ability to detect change.

A potential limitation of this study relates to possible missing data in some criteria. This applies in particular to those criteria that depend upon clinicians recording as a matter of routine the presence or absence of a criterion such as “capillary refill time >2 seconds or other evidence of shock”. As this is a secondary data analysis based on pragmatic recording of routine clinical assessments, the underpinning assumption is that the data recorded on criteria is reasonably complete; however there is no way to verify this. By default, some missing data will be incorrectly attributed to the control group in each analysis. That is, where a criterion is not recorded as being present, that criterion is assumed to be absent. Attempts were made to overcome this by applying criterion definitions to clinical data in other sections to validate and if necessary, update variable values. Using this approach, we were able to impute 20–35% data values, which would have otherwise been missing data. This limitation is common to the whole data set, reflects the reality of clinical practise, and does not preclude fair comparison of the validity and utility of the three tools.

A possible limitation of our study is that we used a complete-case analysis approach. This could bias our results if the data are not ‘missing completely at random’ (MCAR). Multiple imputation is often recommended but it is still based on the assumption that every subject in a randomly chosen sample can be replaced by a new subject that is randomly chosen from the same source population as the original subject, without compromising the conclusions [17]. However, given that the three tools have some common variables in their construction (particularly the ones with missing values), one could still argue that any bias would be non-differential and so our comparison still stands.

This study does not include comparative assessment of the triage tools in the community. The validity and utility of triage tools in the community remains untested.

Morbidity and mortality rates were low during this event when compared to some previous influenza pandemics and the use of anti-viral therapy was generally low in our cohort despite it being widely available at the time. A more severe pandemic may be associated with a greater acceptance of anti-viral therapy and this may impact upon need for higher levels of care and death.

### Generalisability

CATs and PMEWS were developed for use during pandemic events and their criteria address the most likely modes of critical illness arising from influenza, or the complications of influenza. Both were also designed to identify sick patients most likely to benefit from higher levels of care due to other illnesses, which at presentation are indistinguishable from influenza like illness. CATs may have value in other scenarios where high-bar triage is required for both adults and children such as other severe acute respiratory pandemic events and possibly some mass casualty events.

### Conclusions

This study shows that CATs appear better suited as a predictive tool for severe outcomes in pandemic influenza than the CURB-65 score and PMEWS. We propose a CATs score ≥3 as a decision threshold prompting consideration for admission to higher levels of care. This was a retrospective study and the validity and utility of CATs needs to be assessed in a separate prospective cohort including triage in the community. Conducting this study prospectively in a community cohort linked to hospital outcome during a future pandemic would also enable researchers to assess and compare the validity and utility of CATs and other triage tools in relation to hospital admission. Since pandemics are unpredictable and infrequent, limited but potentially useful information would be gained from a prospective evaluation during seasonal influenza periods.

## Supporting Information

Appendix S1 Paediatric (children age <16 years) and Adult Community Assessment Tool referral and admission criteria abridged *from Swine flu clinical package for use when there are exceptional demands on healthcare services* (Department of Health & NHS: 2009).
(PDF)
