# Supplementary material for: Comparison of CATs, CURB-65 and PMEWS as Triage Tools in Pandemic Influenza Admissions to UK Hospitals: Case Control Analysis Using Retrospective Data
Source: PLoS One. 2012 Apr 3;7(4):e34428. doi: 10.1371/journal.pone.0034428 (PMC3317953; doi:10.1371/journal.pone.0034428)
Supplement: Appendix S1 — Paediatric (children age <16 years) and Adult Community Assessment Tool referral and admission criteria abridged from Swine flu clinical package for use when there are exceptional demands on healthcare services (Department of Health & NHS: 2009). (PDF) [file pone.0034428.s001.pdf]

Appendix S1. Paediatric (children age <16 years) and Adult Community Assessment Tool referral and admission criteria abridged *from Swine flu clinical package for use when there are exceptional demands on healthcare services* (Department of Health & NHS: 2009).

Criteria Label	Definitions	
	**Children (age under 16 years)**	**Adults (age 16 years and older)**
**A: Severe respiratory distress**	Lower chest wall indrawing, sternal recession, grunting or noisy breathing when calm.	Severe breathlessness: e.g. unable to complete sentences in one breath. Use of accessory muscles, supra-clavicular recession, tracheal tug or feeling of suffocation.
**B: Increased respiratory rate**	Increased respiratory rate measured over at least 30 seconds. ≥50 breaths per minute if under 1 year, or ≥40 breaths per minute if ≥1year.	Increased respiratory rate measured over at least 30 seconds. Over 30 breaths per minute.
**C: Oxygen saturation ≤ 92% on pulse oximetry breathing air, or on oxygen.**	Oxygen saturation ≤92% on pulse oximetry breathing air *or* on oxygen. Absence of cyanosis is a poor discriminator for severe illness.	Oxygen saturation ≤92% on pulse oximetry, breathing air *or* on oxygen. Absence of cyanosis is a poor discriminator for severe illness.
**D: Respiratory exhaustion**	Respiratory exhaustion or apnoeic episode. Apnoea defined as a ≥20 second pause in breathing.	Respiratory exhaustion. New abnormal breathing pattern, e.g. alternating fast and slow rate or long pauses between breaths.
**E: Evidence of severe clinical dehydration or clinical shock**	Evidence of severe clinical dehydration or clinical shock. Sternal capillary refill time >2 seconds, reduced skin turgor, sunken eyes or fontanelle.	Evidence of severe clinical dehydration or clinical shock. Systolic blood pressure <90mmHg and/or diastolic blood pressure <60mmHg. Sternal capillary refill time >2 seconds, reduced skin turgor.
**F: Altered conscious level**	Altered conscious level. Strikingly agitated or irritable, seizures or floppy infant.	Altered conscious level. New confusion, striking agitation or seizures.
**G: Causing other clinical concern to the clinical team or specialist doctor**	Causing other clinical concern to the clinical team or specialist doctor. E.g. rapidly progressive or unusually prolonged illness.	Causing other clinical concern to the clinical team or specialist doctor. E.g. rapidly progressive or unusually prolonged illness

The community assessment tools are part of a clinical package of care, which includes pathways that provide extra safety measures for people who are moderately unwell, but cannot be admitted to hospital at the time of triage.

These tools and pathways are for use only when high healthcare demand leads to the need for strict hospital admission triage in affected areas. They should not be used when emergency departments and acute admission units are working with their usual establishment of trained staff, and can operate their usual decision pathways, including providing hospital beds for those requiring admission.
